# Plant–Environment Response Pathway Regulation Uncovered by Investigating Non-Typical Legume Symbiosis and Nodulation

**DOI:** 10.3390/plants12101964

**Published:** 2023-05-12

**Authors:** Helen Wilkinson, Alice Coppock, Bethany L. Richmond, Beatriz Lagunas, Miriam L. Gifford

**Affiliations:** 1 School of Life Sciences, University of Warwick, Coventry CV4 7AL, UK; 2 Warwick Integrative Synthetic Biology Centre, University of Warwick, Coventry CV4 7AL, UK

**Keywords:** nodulation, legumes, rhizobia, actinorhizal interactions, stress responses, *Parasponia*

## Abstract

Nitrogen is an essential element needed for plants to survive, and legumes are well known to recruit rhizobia to fix atmospheric nitrogen. In this widely studied symbiosis, legumes develop specific structures on the roots to host specific symbionts. This review explores alternate nodule structures and their functions outside of the more widely studied legume–rhizobial symbiosis, as well as discussing other unusual aspects of nodulation. This includes actinorhizal-*Frankia*, cycad-cyanobacteria, and the non-legume *Parasponia andersonii*-rhizobia symbioses. Nodules are also not restricted to the roots, either, with examples found within stems and leaves. Recent research has shown that legume–rhizobia nodulation brings a great many other benefits, some direct and some indirect. Rhizobial symbiosis can lead to modifications in other pathways, including the priming of defence responses, and to modulated or enhanced resistance to biotic and abiotic stress. With so many avenues to explore, this review discusses recent discoveries and highlights future directions in the study of nodulation.

## 1. Introduction

Nodulation exists in many forms with diverse regulatory inputs and can also significantly vary in its outputs or benefits. Looking beyond rhizobial–legume symbioses, which have been reviewed extensively (e.g., [1]), can help to better understand this dimension of plant–environment interactions. Nitrogen (N), in its many inorganic and organic forms, is one of the most limiting macronutrients for plant growth as it is a vital component of nucleic acids, amino acids, and chlorophyll. These three molecules represent most of the biosynthetic energy investment in plants, and underscore why N availability is so key. Whilst plants can directly take up N from their soil or substrate surroundings themselves, typically via nitrate transporters (such as NRT1.3 [2]) or ammonia transporters (such as AMT2;1 [3]), a subset of plants are able to form a symbiosis with atmospheric N_2_-fixing bacteria to access otherwise unusable N. This includes legume interactions with N_2_-fixing rhizobia, where nodulation occurs to house the rhizobia in times of low N; otherwise, nodulation is inhibited when N is abundant for a wide range of legume species and crops (e.g., [4]). However, there are other plants besides legumes that can form a symbiosis with N_2_-fixing bacteria, such as actinorhizal plant interactions with N_2_-fixing *Frankia*, cycad interactions with N_2_-fixing cyanobacteria, and the five non-legumes within the *Parasponia* genus, each with specificities for both plant and microbe. In addition, whilst the benefits of N provision are typically described regarding their positive impact on growth, there are many other aspects to these relationships that are important to consider. This review first provides a very brief overview of the relatively well-known nodulation that occurs between legumes and rhizobia, before discussing other nodulation relationships and the additional benefits that are gained.

## 2. The (Relatively) Well-Known Rhizobial–Legume Symbiosis of Nodulation

The Fabaceae or Leguminosae family, commonly known as legumes, have more than 22,000 species within 772 genera [5] and a vast amount of them ‘take advantage’ of being able to house N_2_-fixing rhizobia within root-derived structures called nodules. By first considering a “standard” nodulating plant and its symbiotic partners, some context on N_2_-fixing symbiotic partnerships can be provided. Nodulating species can be broadly split into two classes, indeterminate or determinate, with the key difference being whether the nodule has an active meristem or not, respectively. Determinate nodules, which are found in species including *Lotus japonicus*, have cell division that leads to nodule organogenesis occurring in the middle or outer root cortex, and then they lose the meristem early on during development [6]. Indeterminate nodules, such as those in the species *Medicago truncatula*, have cell divisions that lead to nodule organogenesis occurring in the inner cortex and pericycle, and they maintain an active meristem throughout their development [7]. Throughout the nodulation process, from initial plant–rhizobia contact to nodule organogenesis, many different regulatory genes are involved across the different tissue cell types within the root to coordinate rhizobial entry with nodule development. The discussion of these genes goes beyond the scope of this review; [1] provides a recent extensive overview of these processes, and reviews the process of legume nodulation in depth.

**Figure 1:**
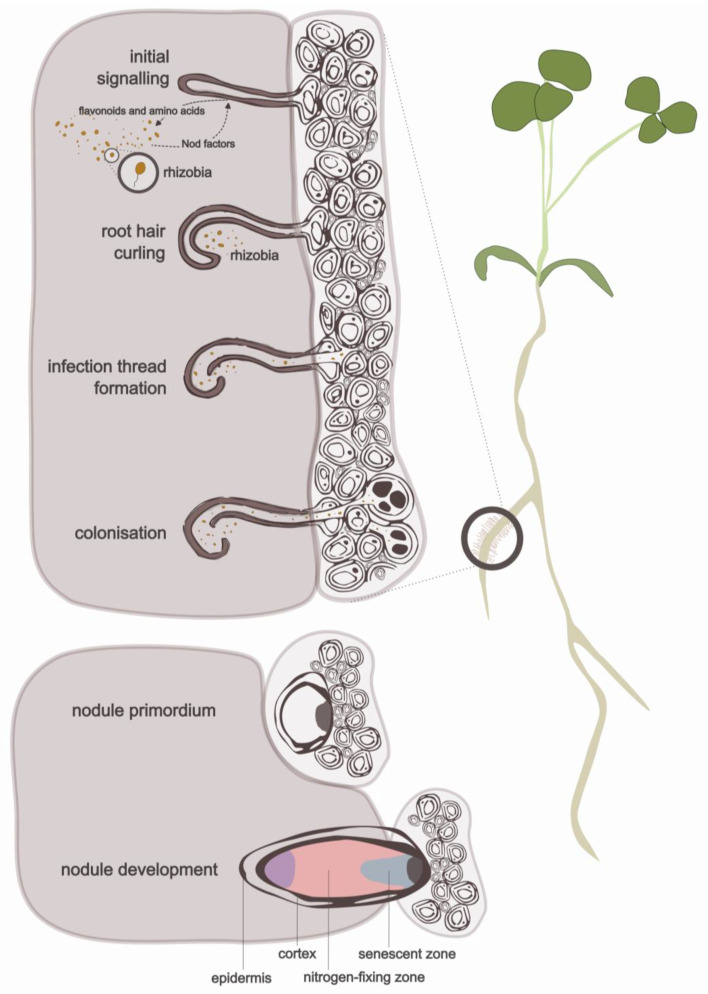
Overview of nodulation in the indeterminate legume *Medicago truncatula.* In the emerging root hair zone, after an initial exchange of signals between plant and rhizobia via plant-derived flavonoids and amino acids, and rhizobia-derived Nod factors, *Medicago truncatula* envelopes the rhizobia via root hair curling. An infection thread forms, allowing controlled entry for the rhizobia towards developing nodule tissue, which is formed concomitantly, acting to house the rhizobia.

Briefly, the process of nodulation begins with signalling between the symbiotic partners, including plant alteration of the defence mechanisms that are usually triggered in response to pathogens. This results in the bacterial symbiont being able to enter the host, through what is mostly a host-controlled process [8] (e.g., for the legume *M. truncatula*, Figure 1). Flavonoids and isoflavonoids are produced via the phenylpropanoid pathway by the plant during times of low N, and released into the surrounding environment [9,10]. The flavonoids and isoflavonoids act as a chemoattractant for the rhizobia; however, it must be noted that there has been recent research suggesting that the importance of flavonoids and isoflavonoids as chemoattractants has been inflated and there may be other attractants such as amino acids that are more important in this process [11]. After a signal being initiated from the plant, the rhizobia can be considered to signal back to the plant with lipochitooligosaccharide Nod factors (NFs). The NF structure varies, enabling species specificity, and NFs are recognised by the host via NF receptor proteins such as *LYK3* and *NFP* in *M. truncatula* [12]. These NFs, however, need to be degraded otherwise rhizobial infection can be delayed [13].

Some legumes can be more specific or more promiscuous with symbiotic partners [14]; however, if a compatible interaction is recognised, the nodulation signalling pathway is switched on [15]. Nodule organogenesis is then triggered, which is a combination of controlled rhizobial entry and division within inner cell types (pericycle and cortex) to generate a nodule primordium. One of the most common entry ways for rhizobia into legumes is via root hair curling, which traps rhizobia, and then the generation of an infection thread through the root hair [16], as seen in legumes including *M. truncatula* [17]. There are also other ways of entry, including a crack-entry system that legumes such as *Arachis hypogaea* (peanut) use [18]; a comprehensive review on intercellular infection, including crack entry, is provided by [19]. Following this, there is a cellular reactive oxygen species (ROS) burst [20], cell wall degradation and membrane remodelling [21], along with cytoskeleton rearrangements [22], that lead to the development of the infection thread towards the emerging nodule.

Once rhizobia are inside the nodule they terminally differentiate into bacteroids if the nodule is of an indeterminate type; if the nodule is determinate, this does not occur [23]. N_2_ fixation in active nodules occurs in both nodulation types, and fixed N_2_ is exchanged for plant-derived carbon and other compounds. The process of forming and maintaining nodules is energy intensive [24], and so selecting for the most efficient symbiotic partner is important, whereby there is maximal gain for both plant and rhizobia. As the nodule becomes older, senescence occurs where the nodule and rhizobia inside are degraded [25]. Nodule senescence can also occur as a means of removing this expensive carbon sink during times of stress, such as drought, but typically only as a last resort to gain the maximum benefit from the nitrogen-fixing rhizobia [26]. Nodule senescence can also be used as a means to control the relationship between host and symbiont: inefficient rhizobia can trigger higher levels of nodule senescence compared to the natural senescence that comes from aging, as observed in *Pisum sativum* (pea) [27].

## 3. Symbiotic Partnerships Outside of Legumes, and Outside of Rhizobia

Whilst N_2_-fixing nodules are typically associated with legumes, there are some exceptions to this whose study is highly informative. The study of these exceptions is particularly useful when viewed hand-in-hand with the knowledge from studying legumes and rhizobia. This diversity of nodulation is vast [28], and this section aims to explore the different symbiotic partnerships found.

### 3.1. Nodulation beyond Legumes: The Example of Parasponia

The *Parasponia* genus contains the only five non-legume species that can form rhizobia-interacting N_2_-fixing nodules [29,30]. *Parasponia andersonii*, found in tropical areas such as Papua New Guinea, is a relatively fast-growing tree that is found on the side of volcanic hills. Remarkably, *P. andersonii* has 290 putative orthologs of *M. truncatula* genes that have enhanced expression in nodules of both *P. andersonii* and *M. truncatula* [31]. Whilst there has been discussion about whether nodulation evolved independently more than once (gain of function many times), or whether it arose once and was lost many times, the existence of orthologs between *M. truncatula* and *P. andersonii* that have similar expression patterns offers some support for a single gain-of-nodulation. If this is the case, studying very close, non-nodulating relatives of *P. andersonii* can be very informative.

*P. andersonii* has been described as relatively promiscuous compared to legumes where there is a high degree of rhizobial partner specificity [32]. There have been mixed findings on whether host promiscuity is advantageous or disadvantageous. It can be considered to be a disadvantage as a promiscuous plant can be described as a “Jack of all trades and master of none”, which can be potentially outcompeted by hosts which have a more specialised and fine-tuned rhizobial partner, which can potentially provide more fixed N_2_ [33,34]. However, promiscuity can also be viewed as an advantage as it can mean there is a higher chance of finding at least one symbiotic partner, enabling wide niche colonisation that the gain of N or other benefits enables [35]. For instance, whilst the legume *Lotus burtii* formed more nodules with a wider variety of rhizobia compared to the legume *L. japonicus*, not all of these symbiotic partnerships had an effect on the shoot length [36]. Whilst [32] demonstrated *P. andersonii* promiscuity, as *P. andersonii* can form nodules containing rhizobia with low levels of N_2_ fixation, there is also evidence to suggest that *P. andersonii* can regulate its symbiotic partnerships. As found in [37], nodules are prevented from forming if a sufficient amount of N is available from the soil.

Unlike the model legumes *M. truncatula* and *L. japonicus*, *P. andersonii* employs a crack-entry system for the rhizobia to enter instead of root hair curling. However, despite this seemingly less sophisticated method of entry, *P. andersonii* still requires the detection of compatible rhizobial NFs [32]. *P. andersonii* also has NF receptors thought to be orthologous to those in legumes such as *M. truncatula*, such as LysM domain receptor kinase *LYK3* [38]. The requirement for NFs and the regulatory control of symbionts suggests a high degree of similarity between *P. andersonii* nodulation and legumes such as *M. truncatula*, on one hand, yet the differing mode of rhizobial entry and promiscuity on the other hand highlights differences that could be investigated to better understand this non-legume nodulator. This information could offer solutions for the transferring of nodulation into other non-legumes.

### 3.2. Nod-Factor-Independent Nodulation in Legumes

Whilst NFs are a key part of the mechanism enabling rhizobial entry in many legumes and *P. andersonii*, there are also legumes that can form nodules with rhizobia without the involvement of NFs. Many of the NF-independent legumes currently being studied belong to *Aeschynomene* spp., which are dalbergoid legumes. These dalbergoid legumes rely on crack-entry for nodulation without the need for NFs. This includes *Aeschynomene indica*, which forms not only root nodules but also stem nodules [39]. A total of 300 *Bradyrhizobium* strains were isolated from *A. indica* nodules, and 19 of these were tested for activity, representing the 19 different haplotypes found. All of these lacked nod genes, and whilst the inoculation of them all led to the formation of root nodules, only the inoculation of six them led to the formation of stem nodules [40]. This suggests that the root nodulation of *A. indica* is more flexible compared to stem nodulation. In *A. evenia*, Nodulation Signalling Pathway 2 (*NSP2*), one of the important nodulation genes found in legumes [41], was also found to be one of the regulators of this NF-independent symbiosis and demonstrated that the conservation of nodulation regulatory machinery is conserved downstream of NFs [42]. It has recently been shown that a different dalbergoid legume, *Arachis hypogaea* (peanut), can also form NF-independent nodules, although less efficiently, despite previously being considered to be NF-dependent [43]. The way that this NF-independent symbiosis can occur has been found to involve the effectors secreted through the bacterial type III secretion system, in a mode that can be considered to hijack or co-opt the nodulation signalling pathway [44,45]

These recent studies have highlighted that not all legumes are restricted to NF signalling, and it would be interesting to see if *P. andersonii* can also form NF-independent nodules, since it uses crack-entry similarly to *Aeschynomene* spp.

### 3.3. Symbiotic Partnerships in Non-Rhizobial Nodules

Actinorhizal plants consist of eight families and twenty-five genera, including *Hippophae rhamnoids*, *Datisca glomerata*, and *Casuarina glauca*, that are able to form N_2_-fixing nodules with *Frankia* bacteria rather than rhizobia [46]. Remarkably, this relationship is estimated to account for as much as 15–25% of global N_2_ fixation [47,48]. Both *C. glauca* and *H. rhamnoids* could be considered as model species, and the generation of a reference genome for *H. rhamnoids* in 2022 [49] and an updated version of the *C. glauca* genome published in 2023 [50] is enabling some progress to be made on elucidating the molecular mechanism of actinorhizal interactions. *Frankia* bacteria are Gram-positive, branching, filamentous soil bacteria [51], many of which have the ability to form a symbiosis with actinorhizal plants [52]. *Frankia* share many similarities with rhizobia in terms of N_2_-fixation, as they both produce nitrogenase [53] and can invade via infection threads [54]. However, they differ from rhizobia in that they can also fix N_2_ in aerobic conditions outside of the roots of plants. They can do this due to the presence of vesicle structures at the ends of their hyphae, which protect the nitrogenase from oxygen [55,56]. Nodulation is a host-controlled process which is seen in relation to both *Frankia* and rhizobia relationships with their own hosts; actinorhizal plants for *Frankia* and legumes for rhizobia, suggesting there are also host regulatory similarities [57]. 

The signalling machinery that *Frankia* uses is still being unravelled, with some *Frankia* having nod genes and others not. Purified rhizobial NFs do not themselves trigger a response in actinorhizal plants [58], highlighting distinctions between rhizobial- and *Frankia*-interacting mechanisms. The proteomic analysis of *Frankia* responses to root exudates from compatible and incompatible actinorhizal plants uncovered a greater response when the plant was compatible, helping to identify the mechanism underpinning an efficient interaction [59]. Furthermore, in the actinorhizal plant *C. glauca*, Root Hair Deforming Factor (*RHDF*) and the *NIN* activating factor (*NINA*) were highly expressed in response to compatible *Frankia* or closely related *Frankia* strains, but not incompatible strains [60]. It has also been recently found that the biphenyl-type diarylheptanoid alunsonol could function as a signal from actinorhizal plants to *Frankia* [61]. Whilst the signalling mechanism between actinorhizal plants and their bacterial symbionts is still being investigated, there is exciting research being conducted on how it functions, and this can be usefully compared with rhizobial nodulation mechanisms in legumes.

### 3.4. Plant–Microbe Symbiosis without Root Nodules

Cycads employ a symbiotic relationship with cyanobacteria and host them within their roots instead of forming nodules. As reviewed in [62], cycads are the oldest extant seed plant and offer an ancient and other non-legume example of a N_2_ fixing relationship. These ancient plants once dominated forests across the globe, after they evolved around 300 million years ago, but are considered to have been ‘ousted’ from that dominating top spot by ‘higher’ plants. Currently, all known species of cycads, such as *Cycas revoluta*, also known as sago palm, a woody plant that originated in Southeast Asia [63], have been found to form endophytic relationships with photosynthetic cyanobacteria which are housed in coralloid roots, as reviewed in [64]. The cyanobacteria live in a cortical cell layer termed the cyanobacterial zone, which can be seen as a green ring when the coralloid root is cut open [65]. *C. revoluta* tends to be found in nutrient-poor soils such as on coastal cliffs, and so the advantage of a symbiotic partner supplying N can be hypothesised to have enabled them to colonise a challenging environment [66].

Cyanobacteria are multicellular bacteria that can photosynthesise as well as fix N_2_. Whilst this seems contradictory, as nitrogenase is inactivated by oxygen, cyanobacteria separate these two systems spatially, through cellular differentiation (by creating a heterocyst for N_2_ fixation to occur) [67] or temporally, via a biological clock [68]. Cycads use cyanobacteria chemoattractants, which are referred to as hormogonium-inducing factors, and include molecules such as diacylglycerol 1-palmitoyl-2-linoleoyl-sn-glycerol [66]. Once cyanobacteria are inside the plant, the plant produces hormogonium-repressing factors which allows for the heterocyst to form [66]. Aside from the involvement of chemoattractants, we still have much to learn about the signalling mechanism between cyanobacteria and cycads, as explored in another review [64].

A recent discovery highlights another potentially ancient symbiosis between N_2_-fixing bacteria and *Posidonia oceanica*, a highly productive Mediterranean seagrass [69]. The roots of N_2_-fixing *P. oceanica* plants were found to harbour an abundant population of a novel *Celerinatantimonas sp.* Of bacteria, *Candidatus Celerinatantimonas neptuna*, with direct evidence of the transfer of up 98% of fixed N being transferred to the plant [69]. These bacteria were identified throughout the root cortex, housed both intercellularly and within the root cells [69]. Due to the recent nature of their discovery, the mechanism of signalling with this aquatic seagrass is currently unknown, but this symbiosis is of interest in the context of pollution within the ecosystem. *P. oceanica* also hosts N_2_-fixing epiphytes on the surface of the leaves, which are exposed to contaminants found within the ocean, such as the chemicals in sunscreen [70]. Investigating whether root symbionts, which are relatively protected within the root, enable aquatic plants to thrive in polluted situations is a pressing question.

Another structure that we can learn much from is aerial roots which are found on some orchids and are thought to play a role in nutrient uptake and even photosynthesis [71,72]. There are some species of orchids that interact with cyanobacteria housed within the cracks of their aerial roots, which could provide fixed N_2_ for the host, although this needs to be further studied [73]. There is also evidence that maize brace roots, a type of aerial root, can accommodate N_2_-fixing bacteria within mucilage exuded by the aerial roots that can provide as much as 82% of the plant-required N [74]. Understanding the mechanism by which these symbiotic relationships arise could help make use of beneficial microbes in agriculture and the environment.

## 4. Extra Bonuses of Hosting Microbes

The occurrence of different types of N_2_-fixing relationships in plants show us that nodulation is not the only mode of plant–microbe N-interaction. Reciprocally, we also know that symbiotic bacteria can provide more than just N to plants.

### 4.1. Defence Priming

**Figure 2:**
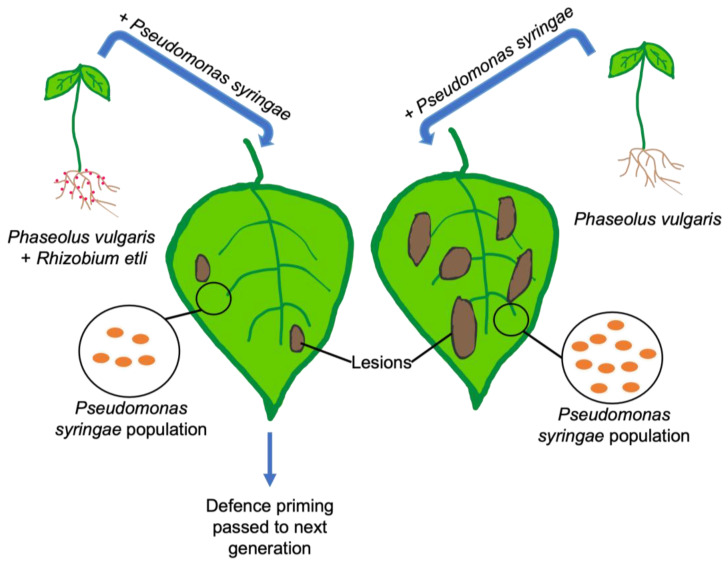
Rhizobium etli inoculation primes Phaseolus vulgaris resistance to Pseudomonas syringae infection. After the inoculation of Phaseolus vulgaris (common bean) with Rhizobium etli, fewer lesions and a lower amount of pathogenic Pseuodmonas syringae bacteria were found after infection. This defence priming was also found in the next generation.

Plants that house symbiotic bacteria have to walk a delicate tightrope of allowing these bacteria in, at the same time as keeping pathogens out. However, the interaction is even more complicated since it has been found that symbiotic bacteria can provide the priming of defences for its host plant. Defence priming can include different aspects, including the accumulation of salicylic acid (a phytohormone key to the defence response [75]) and enabling plant defence genes to be activated more quickly in the face of subsequent pathogen interaction. For instance, it was found by [76] that *Rhizobium etli* protected *Phaseolus vulgaris* L. (common bean) from *Pseudomonas syringae* (Figure 2). In this example, the bacterial population of *P. syringae* within the leaf tissue was halved in plants inoculated with *R. etli* in comparison to the uninoculated control, and the lesions caused by the pathogen in the leaf were 75% smaller in inoculated plants. Not only did *R. etli* protect *P. vulgaris* when directly inoculated, this defence priming was also passed down to the next generation. Unlike the parent plants, the next generation had not been exposed to *R. etli*, yet the plants still exhibited an about three-fold decrease in the abundance of *P. syringae* in the leaf tissue and approximately an 80% reduction in the size of the lesion tissue formed in comparison to the control. *M. truncatula* and *P. sativum* have also been found to benefit from symbiotic bacteria defence priming. When inoculated with *Sinorhizobium meliloti* and *Rhizobium leguminosarum*, the amount of free salicylic acid in the plant shoots increased in comparison to uninoculated plants by approximately 10% in *M. truncatula* and 30% in *P. sativum* when exposed to *Erysiphe pisi* (mildew fungus), increasing the resistance to the fungus [77].

Alongside root nodules, a structure that has also been linked to potentially increasing defence responses are leaf nodules. These have been reported in less than 500 species and their size and shape differs from species to species. One example of a plant capable of forming leaf nodules is the dicot *Psychotria kirkii*, housing its endosymbiont *Burkholderia* sp. [78]. Whilst these endosymbionts are not associated with N_2_-fixation, they have been suggested to provide the host with secondary metabolites to aid in defence [79,80]. The symbionts are transmitted through the seeds; however, some bacteria do enter though leaf stomata openings, as reviewed in [81]. This is a very specialised form of symbiosis which is so tightly integrated that the plant and bacteria cannot survive without each other; to cultivate the leaf bacteria outside of *P. kirkii* has thus far proven unsuccessful [78]. However, genome analysis of the *Burkholderia* sp. Within the leaf nodules has been able to be conducted and genome reduction has been found to be a common trait amongst them [82]. Recent work has also established an alternate leaf nodule symbiosis model between the monocot *Dioscorea sansibarensis* and *Orrella dioscoreae*, where it has been possible to culture the host and symbiont independently of one another. The bacterial symbiont *O. dioscoreae* also produces secondary metabolites, but the exact role of them is as of yet undetermined. However, these secondary metabolites could have a similar role in aiding the plant’s defence system, like the *Burkholderia* sp. symbiosis with *P. kirkii* [83]. The development of these leaf nodule systems could enable further investigation into how these different forms of nodulation may be able to aid the plant defence response, perhaps acting as a valuable comparison to the defence priming related to root nodulation, as described earlier. 

Not only can rhizobia prime their hosts against microbial pathogens in a number of cases, but they have also been found to potentially offer anti-herbivory features. In [84], differing levels of *Bradyrhizobium japonicum* inoculation of *Glycine max* (soybean) were carried out, with plants also grown on different levels of soil N; high soil N and high rhizobial inoculation led to similar amounts of plant % total N. After 5 weeks of growth, plants were then infected with *Halicoverpa zea* (soybean podworm). It was found that the larvae preferred the plants with lower rhizobial colonisation, despite all plants having similar total N levels and regardless of the origin of the N (taken up from soil or fixed via rhizobia). This was also surprising as *H. zea* larvae had higher growth rates in plants with high levels of rhizobial inoculation (0.881 ± 0.059 g/g/day) compared to high soil N (0.677 ± 0.051 g/g/day). This could potentially be due the rhizobial-related fixed N, but also other rhizobial plant growth promotion activities leading to increased nutritional quality, such as carbohydrates and phosphates in the plant. Such improvements in plant nutrition have been found for *Rhizobium radiobacter* inoculation of lettuce [85].

**Figure 3:**
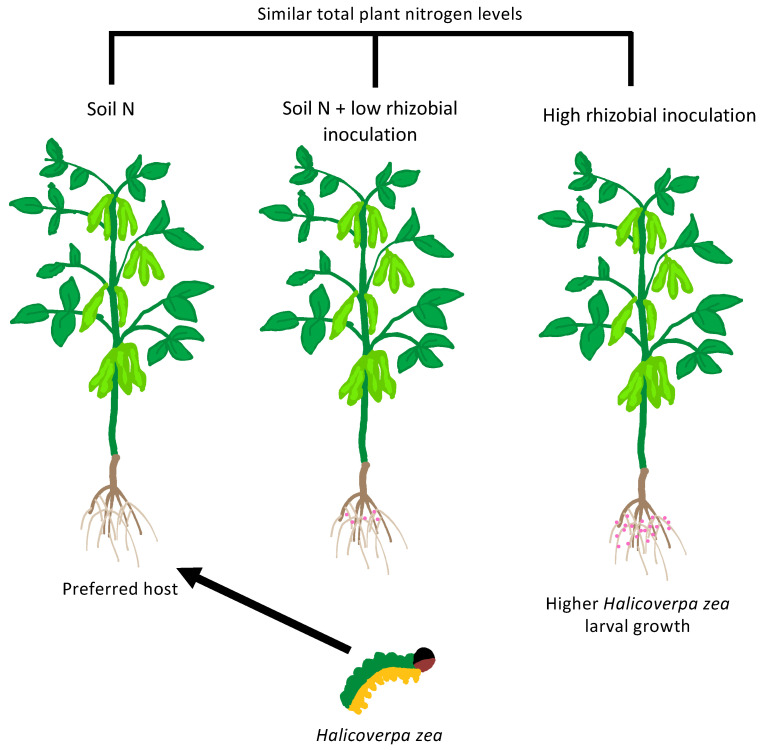
Rhizobia confer protection to Glycine max from Halicoverpa zea. Despite Halicoverpa zea larvae growing larger on plants with a high amount of rhizobial inoculation, when offered a choice H. zea chooses plants with the lowest rhizobial load.

This observation, that *H. zea* preferred high soil N compared to conditions with high rhizobial inoculum, was found to be linked to the defence-related hormone jasmonic acid being found to be produced to a higher level in plants inoculated with larger amounts of rhizobia (Figure 3). It would be interesting to discover if promiscuous plants are able to better defend themselves against herbivores, due to their ability to interact with a larger number of rhizobial species, as a possible evolutionary advantage of entering in widespread interactions.

Increasing plant defence against pathogens via the use of rhizobial inoculants in plants could also help replace the use of harmful pesticides if we can better understand their protectant function and range, as reviewed in [86]. This could not only offer a benefit for crops that already form nodules with N_2_-fixing microbes, but for crop varieties that may be developed as the research to transfer nodulation ability to new crops develops, informed by research on species such as the non-legume *P. andersonii* as well as understanding of leaf nodules and aerial roots housing N_2_-fixing bacteria. Whether the plant and microbe need to associate directly in order to bring about this biotic protection, or if plant pathways simply need to be activated (e.g., by NF addition), is a highly relevant question if we are to use this information in crop protection.

### 4.2. Abiotic Stress Resistance

Not only do plants face threats from biotic stress, including pathogens and herbivores, but there are also many abiotic and environmental stresses such as drought, high temperature, flooding, and high salt levels. Drought has been shown to reduce cereal production by 10% on average across the globe [87], and droughts will become more common as the climate changes. Under drought, salt excess can become a major issue as it builds up in the soil and can change the osmotic balance, with resultant salt stress being shown to reduce yields in crops such as rice [88]; flooding also causes major crop losses and reduced yields [89]. Exploring how rhizobia can help plants mitigate the effects of abiotic stress is therefore highly relevant in developing sustainable crop production in the face of a warming planet. It has been found that N_2_-fixing bacteria can indirectly provide drought stress tolerance to their hosts. By providing extra N, symbiotic bacteria such as *Mesorhizobium huakuii* can allow for plants such as *Astragalus sinicus* L. to produce more arginine, which is linked to drought stress tolerance [90]. However, it goes further than just providing extra N; nodulating *M. truncatula* has been shown to recover from drought faster compared to when it is not being nodulated. The rhizobia within the nodules may aid the plant through the period of drought by delaying the senescence of the leaves by allowing the accumulation of the osmolyte proline by the plant [91]. It has also been shown that nodulation with different rhizobial strains can result in different performances of *Glycine max* under drought stress. Rhizobial strains showing higher osmotic stress resistance outside of symbiosis were found to have the highest antioxidative parameters and more osmotic stress tolerance in the host [92].

Salt-tolerant rhizobia have been shown to provide better drought resistance in *Phaseolus vulgaris* (common bean); the shoot dry weight was almost 30% higher in plants inoculated with a more salt-tolerant rhizobia compared to plants inoculated with lower salt-tolerant rhizobia after 7 days of drought [93]. This indicates that rhizobial salt tolerance properties can influence how well the plant host can survive higher salt levels. This has also been shown to be the case with *Vicia faba* (faba bean), where rhizobia strains with different levels of salt tolerance could lead to increased salinity tolerance for the host. Rhizobia with higher salt tolerance themselves had on average higher N_2_ fixing capacity (32.28 ± 1.47 µmol h^−1^Plant^−1^) compared to rhizobia with a lower salt tolerance (13.83 ± 1.65 µmol h^−1^Plant^−1^), and testing these under salt conditions may help *V. faba* survive these conditions [94]. Salt-tolerant rhizobia have also been shown to directly improve the salt tolerance of plants by changing amino acid composition towards the accumulation of amino acids with protective functions in the nodules, such as proline, which is involved in osmoregulation [95,96]. Sulphur (S) is an important macronutrient and a major component of thiols, which act as antioxidant protectants, protecting plants from reactive oxygen species damage caused by stress, such as high levels of salt, as reviewed in [97]. It has been found that nodules could be important in S-assimilation and thiol biosynthesis, and that the bacteria within the nodules are important in this process as genes for S-uptake and metabolism are upregulated in rhizobia within nodules. For example, when *L. japonicus* was inoculated with *Mesorhizobium loti* it was found that the nodules were rich in these protective thiols [98]. Whilst there are mechanisms in legumes to optimise the efficiency of N_2_-fixing symbioses for N update, if rhizobia are providing more than just N, it is likely that there other regulatory signalling mechanisms involved.

By employing rhizobial nodulation under particular environmental conditions, *Sesbania rostrata*, a tropical, semi-aquatic legume, is able to withstand high levels of salinity as well as flooding. Under aerobic conditions, root nodules can be infected via root hair infection threads [99]. It has been found that the presence of rhizobia stimulates the activity of enzymes that protect against ROS damage caused by high levels of salt [100]. Under flooding, *S. rostrata* can form N_2_-fixing nodules on the roots or on the stem at dormant root primordia, which are structures that can develop into roots when submerged, with the rhizobia entering via a crack-entry system under flooded conditions [101]. This demonstrates a flexibility in the relationship between *S. rostrata* and compatible rhizobia. However, *S. rostrata* may recruit N_2_-fixing symbionts even before germination to create a biofilm of compatible rhizobia ready for infection. *S. rostrata* seed exudates have been found to increase the expression of the chemotaxis pathway and flagella synthesis in the compatible rhizobial strain *Azorhizobium caulinodans* ORS571 [102]. As *S. rostrata* is also a crack entry legume, it would be interesting to determine if it can be NF-independent, like *A. hypogaea* [43]. Both stem and root nodules have similar nitrogenase activity, which shows that *S. rostrata* gains an advantage of fixed N_2_ in both non-flooded and flooded conditions [103]. Learning more about stem nodules can help the host to overcome environmental issues such as flooding, which would therefore help in understanding how to mitigate climate-related abiotic impacts including flooding [104], drought [105], and salt [106].

## 5. Conclusions

**Figure 4:**
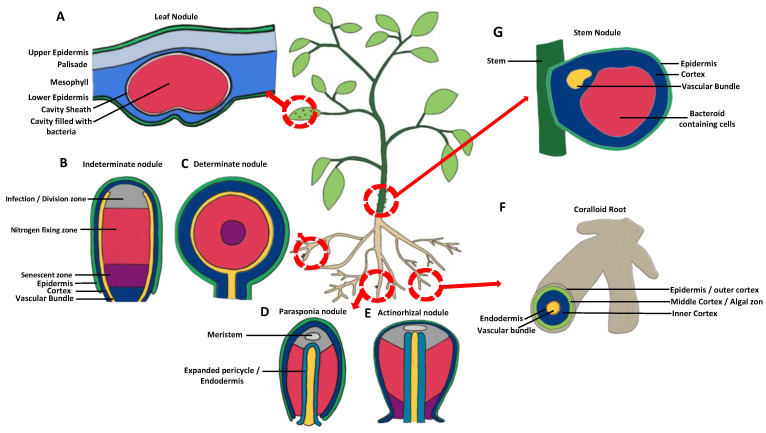
Nodule diversity: form and function. An overview of the different types of nodules mentioned throughout this review: (**A**) Leaf nodules such as those found on *Psychotria kirkii* may not provide fixed N but can bring defence-priming benefits. Figure adapted from original microscopy image [81]. (**B**) Indeterminate nodules are found on legumes such as *Medicago truncatula*. Figure adapted from [107]. (**C**) Determinate nodules are found on legumes such as *Lotus japonicas*. Figure adapted from [86]. (**D**) One of five non-legumes which forms nodules with rhizobia, *Parasponia andersonii*. Figure adapted from original microscopy image [108]. (**E**) Actinorhizal nodules found on actinorhizal plants that house N_2_-fixing *Frankia* bacteria. Figure adapted from original microscopy image [109]. (**F**) Coralloid roots as found on the cycads, which have a green ring (algal zone) where the cyanobacteria are found. Figure adapted from original photo [64]. (**G**) Stem nodules such as the ones found on *Sesbania rostrata* and *Aeschynomene* spp. Figure adapted from original microscope image [110].

A symbiotic relationship has two partners, in this case the host plant and its symbiotic bacteria within the nodules. It is important to understand the reciprocal benefits in order to understand the relationship fully. Our understanding that the rhizobia can provide more to its host than just N can inform future research into mitigating abiotic and biotic stress, bringing benefits of reduced pesticides as well as reduced fertiliser use. To better identify these beneficial directions, it is also important to consider a much wider range of species (and locations) in the investigation of beneficial bacteria-harbouring interactions (Figure 4).

Studying different forms of nodulation and different symbionts to uncover mechanistic links between plant processes is also an important avenue to research as these species interactions have been found in extreme environments, such as the case of cycads and their hosted cyanobacteria. Another interesting direction would be to investigate *P. kirkii* and its endosymbiont, if they can ever be successfully separated, and to see if *P. andersonii* could utilise NF-independent signalling in a similar manner to *Aeschynomene* spp. With updated genomes becoming available for actinorhizal plants as well as increasing numbers of accessions of the species described above, the molecular machinery behind actinorhizal plants and *Frankia* can continue to be unravelled. There are many avenues of research to explore, and potential links in the modulation of plant–microbe symbiotic relationships that are exploited by plants or microbes, that we can use to improve plant growth and protection and better characterise how plants and microbes work together.

## Data Availability

No new data were created for this review article.
